# Effect of Godelieve Denys-Struyf (GDS) muscle and articulation chain treatment on clinical variables of patients with chronic low back pain and lumbar disc degeneration: a pilot feasibility randomized controlled trial

**DOI:** 10.1186/s40814-023-01268-4

**Published:** 2023-03-17

**Authors:** Sidsel Lombardo, Gunvor Hilde, Milada Cvancarova Småstuen, Margreth Grotle

**Affiliations:** 1 Physiotherapy Department at Vestfold Hospital Trust (VHT), P.O. Box 2168, 3103 Tønsberg, Norway; 2 Center for Intelligent Musculoskeletal Health, Department of Rehabilitation Science and Health Technology, Oslo Metropolitan University, P.O. Box 4, St. Olavs plass, NO-0130 Oslo, Norway; 3 Department of Research and Innovation, Division of Clinical Neuroscience, Oslo University Hospital HF, Ulleval, Bygg 37b, P.O. Box 4956, Nydalen, 0424 Oslo, Norway

**Keywords:** Exercise therapy, Feasibility studies, Intervertebral disc degeneration, Low back pain, Physical therapy modalities, Randomized controlled trial, Rehabilitation

## Abstract

**Background:**

Patients with chronic low back pain (LBP) and lumbar disc degeneration are recommended to try out nonsurgical treatment options before surgery. There is need for good nonsurgical alternatives that can be adapted to the patient’s needs and level of function. The aim of this pilot trial was to test study feasibility of a future full randomized controlled trial (RCT) evaluating the feasibility and benefit of the physiotherapy-based Godelieve Denys-Struyf (GDS) muscle and articulation chain treatment for patients with chronic LBP and lumbar disc degeneration referred to surgical assessment in a hospital outpatient clinic.

**Methods:**

This study is a single-center, two-arm, single-blinded, pilot RCT conducted in a regional hospital in Norway. Patients of age 35–75 years with chronic (> 3 months) LBP and degenerative lumbar disc(s) verified by imaging were included. They filled in a baseline questionnaire prior to randomization, including the Oswestry Disability Index (ODI), numerical rating scale for pain in back and pain in leg, and the EuroQoL 5L. Patients in the control group were free to use treatment as usual. Patients in the intervention group received 8 sessions over a period of 10 to 14 weeks of GDS muscle and articulation chain treatment.

**Results:**

The recruitment rate was slow, approximately 3/4th of the referred patients met the inclusion criteria, but majority of eligible participants (94%) were willing to participate. A total of 30 patients were randomized into the two groups. The randomization led to skewed distribution of radiating leg pain in the two groups. All participants except one (97%) completed 4 months follow-up. No serious adverse events attributable to the trial treatments were reported. The Oswestry Disability Index (ODI) and leg pain intensity scale were both suitable as primary outcomes in a full trial. The mean change in the ODI score was 8.7 (*SD* 16.1) points in the GDS arm, whereas there was a minor deterioration in the ODI scores of −3.7 (7.5) points in the control arm. A sample size calculation based on the ODI scores resulted in a number needed to treat of 3.

**Conclusions:**

A future full RCT is feasible and would provide evidence about the effectiveness of a GDS treatment for patients with chronic LBP and lumbar disc degeneration.

**Trial registration:**

ClinicalTrials.gov ID: 910193.

**Supplementary Information:**

The online version contains supplementary material available at 10.1186/s40814-023-01268-4.

## Key messages regarding feasibility

- **What uncertainties existed regarding the feasibility?** The most important uncertainty is the duration of recruitment of patients to a full-scale trial.
- **What are the key feasibility findings?** This pilot trial showed that feasibility worked well in terms of eligibility criteria, patient information, processes for consent and randomization, follow-up rate, treatment outcomes, treatment protocol, and compliance to the GDS intervention. The recruitment rate was slow, however.
- **What are the implications of the feasibility findings for the design of the main study?** In a future trial, it is important to get a more efficient recruitment in place and to stratify for radiating pain to buttock and/or leg.

## Background

### Chronic low back pain and intervertebral disc degeneration

Chronic low back pain (LBP) is characterized by persistent and/or recurring pain in the back and is often associated with neurological symptoms in the lower limbs [1]. In aging people, degenerative changes in the intervertebral disc, such as spinal stenosis with or without degenerative spondylolisthesis, are commonly observed by imaging techniques with prevalence estimates as high as 57% (95% *CI* 55–60) in patients with LBP [1, 2]. An increasing amount of patients with chronic LBP and intervertebral disc degeneration are referred for surgical treatment, which may take the form of either fusion or decompression of nerve roots [3]. Surgery always comes with higher costs and greater risks of adverse events as compared to conservative treatment options such as multidisciplinary rehabilitation, which has shown similar effectiveness as surgical treatments [4, 5]. In cases with spinal stenosis, the effectiveness of surgery versus conservative treatments might be better, but the evidence is inconclusive [6, 7]. Conservative treatment modalities are often recommended as first-line treatments, and these typically are graded activity or exercise programs that target improvements in daily functions taking individual needs, preferences, and capabilities into account [3]. For patients who do not respond to first-line treatments and who are substantially disabled by pain, an active approach might be combined with cognitive behavioral therapy [7–9] and passive modalities such as spinal mobilization, massage, or acupuncture [10].

Motor control exercises as treatment modality for chronic low back pain have gained popularity in physiotherapy practice, which is based on several randomized, controlled trials during the last two decades showing promising effect when treating patients with chronic low back pain [11, 12]. Motor control exercises focus on the activation of the deep trunk muscles and target the restoration of activation and coordination of these muscles. In a systematic review from 2016, there was low to moderate quality evidence that motor control exercises have a clinical effect for improving pain and disability at short-, intermediate-, and long-term follow-up when compared with a minimal intervention for patients with chronic low back pain [13]. However, this systematic review also concludes that motor control exercises are not superior to other forms of exercises. Therefore, they recommend that the choice of exercise for chronic low back pain should depend on patient or therapist preferences.

Motor control exercise methods are numerous and might vary slightly across nations. One frequently used method in France, Belgium, and Mediterranean countries is named the Godelieve Denys-Struyf (GDS) muscle and articulation chain method. It was developed in the seventies by the Belgian Physiotherapist Godelieve Denys-Struyf. It has since then been further developed in Belgium and France, with the French Physiotherapist Philippe Campignion as a main contributor and author. The GDS method classifies all muscles, including those influencing lumbar-pelvic and spinal stability, into six muscle chain groups, according to their anatomy and role in postures and movements. It builds on the assumption that balanced tension and activation across these muscle chains contributes to adequate neuromuscular, biomechanical and psychomotor control, whereas unbalanced tension across them may explain the presence of pain, as subacute or chronic low back pain LBP. The aim of GDS treatment is to obtain balance between tonus/activity in the different muscle chains and reprogram certain movements in order to achieve optimal motor control. To our knowledge, two former randomized controlled studies have evaluated the effect of GDS treatment for LBP [14, 15]. Diaz-Arribas et al. from 2009 compared 15 GDS sessions to 15 sessions of conventional physiotherapy among 137 patients with nonspecific chronic low back pain searching primary care. After 3 and 6 months, the GDS intervention group showed significantly larger improvements in pain, function, and quality of life as compared to the control group [14]. A cluster randomized trial from 2015 included 461 patients with subacute or chronic LBP [15]. They received either GDS sessions by group or individually or control treatment (as usual). The results showed that GDS provided in group sessions improved function significantly more than the two other groups, but the effect was small. There has been no publication about GDS treatment for patients with chronic LBP with additional verified intervertebral disc degeneration.

The aim of this paper is therefore to report a pilot randomized controlled trial (RCT) to test the feasibility of a future, full-scale trial to evaluate the effectiveness of a GDS treatment as compared to treatment as usual for patients referred to a surgical assessment by an orthopedic specialist. The specific objectives were to evaluate feasibility in terms of (a) process of recruitment, including willingness of participants to be randomized; (b) selection criteria for a full-scale trial process of recruitment, the randomization procedure, and follow-up rates; (c) participants experience of and compliance to GDS treatment; and (d) outcome measures, including estimate the variability of outcomes in this patient population and calculate sample size for a full-scale trial.

## Methods

This pilot randomized controlled trial is reported in line with the CONSORT 2010 extended guidelines to randomized pilot and feasibility trials [16].

### Trial design and setting

This pilot trial was a single-center, two-arm, single-blinded pilot RCT with a treatment phase of 10 to 14 weeks (4 weekly sessions, then some more spaced) and follow-up around 4 months after inclusion. The trial was performed in accordance with the Helsinki Declaration and the International Conference on Harmonisation of Good Clinical Practice, and was registered at ClinicalTrials.gov in June 2020 under the identifier NCT910193. The Regional Committee for Medical Research Ethics South-East Norway (2017/2547/REK sør-øst) approved the pilot trial before it started. The study was conducted at the Department of Physiotherapy, Vestfold Hospital Trust (VHT), Norway, and was funded by the hospital. Researchers at the Department of Physiotherapy, Oslo Metropolitan University, were responsible for design, allocation procedure, and methods for this pilot trial. All participants gave written informed consent before entering the study.

### Participants

The participants were included according to the following criteria: (i) age 35–75 years, (ii) willing and able to participate, (iii) chronic (> 3 months) low back pain, and (iv) degenerative disc(s) in the lumbar spine verified by imaging (2022 ICD-10-CM Diagnosis Code M51.36). Participants were excluded according to the following criteria: (i) severe psychiatric disorder, (ii) comorbidity that prevented the patient from performing exercises and gradually increase general activity when back/leg function allowed it, (iii) undergone spinal fusion or referred to spinal surgery, (iv) pregnancy, and (v) in a process of applying for disability benefits/compensation due to back pain.

### Identification and recruitment

The study participants were referred from general practitioners (GPs) in Vestfold County to a specialist in orthopedic surgery or specialists in physical medicine at VHT for an examination and assessment with respect to surgical treatment or not. If the referred patients were considered inoperable or wanted to postpone surgery, they were referred further to the project staff at the Department of Physiotherapy at VHT, where they were informed about the study and screened for eligibility criteria. Participants who were willing to participate received a full participant information sheet and consent form. After filling in the baseline questionnaire, the participants were sent home and informed that they would be contacted regarding the treatment allocation within the next day. The participants had the opportunity to withdraw at any time, without any consequence for the person’s further health services or opportunity for ordinary treatment.

### Randomization

Eligible participants who gave written informed consent to participate were randomized in a 1:1 ratio. A statistician (MZS) at the Musculoskeletal Health Research Group (MUSKHealth.com) at OsloMet was responsible for the randomization sequence. A collaborator in the project staff (SS) contacted (by telephone text message) the statistician at OsloMet for the allocation code and directly informed the participant about their allocated treatment.

### Blinding

In this study, we could not blind the participants with respect to what treatment they got nor the treating physiotherapist (SL). However, the project collaborator (SS) who administered the information regarding treatment allocation, and the posttreatment questionnaire after 4 months, was blinded with respect to treatment allocation. In addition, analyses of patient-reported outcomes were conducted and verified by the blinded statistician.

### Sample size

This pilot study aimed to explore the methods proposed to conduct a full-scale trial and not to detect a true difference between treatment groups. In this context, we relied on a recommendation of at least 12 participants per group as a rule of thumb for pilot studies [17]. Taking into account potential dropout of participants, we decided to include 30 participants for this pilot study, as an external pilot trial interim analyses and stopping rules were not required.

### Interventions

The participants in the intervention group were examined and treated according to the principles of the GDS method. We aimed to understand the patient’s nature and muscular patterns, unravel tensions that hinder natural body movement in order to stimulate more functional movement patterns for ergonomic body use. Together with the patient, we proposed a treatment program. In line with the GDS method, we applied techniques such as various stretching and respiration exercises, massages, mild manipulations, and movements for good function, all adapted to each patient’s characteristics and needs. The patient was also encouraged to increase their body awareness and to perform tailored home exercises that should typically be effectuated for 15 to 20 min once or twice a week. The home exercises consisted of stretching of contracted muscle chains, ergonomic movements, and respiration exercises, recommended to be done once or twice a week. They received up to 8 individual treatment sessions enduring approximately 1 h, including the baseline examination. The patients paid a minor fee for the treatments, 50% of the normal physiotherapy rate in terms of price. The GDS treatment was administered at the hospital outpatient clinic.

The control group received standard treatment from their GP, possibly referred to physiotherapy, chiropractor, or whatever they preferred. Type of treatment received in the follow-up period was recorded in the follow-up questionnaire.

### Data collection

Baseline data collection consisted of a baseline questionnaire, which was administered prior to randomization. Patient-reported outcomes were assessed approximately 4 months after treatment allocation and were sent to patients per mail with a stamped envelope for return. Patients who did not respond were reminded twice. All data collected on paper was transferred to an EpiData program at VHT.

The baseline questionnaire consisted of information regarding sociodemographic background variables and standardized outcome measures. Background variables concerned age, gender, level of education (primary and high school, college or university < 4 years, or university education of 4 years or more), smoking (yes/no), marital status (married, cohabitant, single), employment status (employed, sick leave, disability pension, age pensioned, unemployed), pain localization (back pain, radiating pain to buttocks and/or legs), sensibility changes in back/buttocks/legs), former surgery (yes/no/fixation?), pain duration (< 3 months, 3–12 months, 12–24 months, > 2 years), and use of pain or sleep/relaxation medication weekly or more (yes/no). In order to describe the patients risk of persistent disabling pain, the STarT Back screening questionnaire was used [18].

Four patient-reported outcome measures were included. The primary outcome, functional disability due to low back pain, was assessed by using the Oswestry Disability Index (ODI) [19, 20], version 2.0. This questionnaire assesses and has ten different sections. The first section assesses pain intensity, and the following nine sections assess how back or leg pain is affecting the patient ability to manage activities of daily living. The score for each section is rated from 0 to 5, and the highest possible score for all sections is 50. The patient’s score is then transferred into a percentage score ranging from 0 (no) to 100% (maximum pain-related disability). Secondary outcomes were pain intensity, respectively in back and leg, assessed on a visual analogue scale ranging from 0 to 100 [21] and health-related quality of life measured by the EuroQoL 5L (EQ-5D-5L) [22]. The EQ-5D-5L exhibits excellent psychometric properties across a broad range of populations, conditions, and settings [23].

For each participant, the treating physiotherapist recorded full details of the treatment period, such as number of treatment sessions, any nonattendance, and any adverse events. Number of treatment sessions was used a measure of compliance (or adherence). We did not expect serious adverse events in relation to the treatment, except minor soreness in the muscles after the initial sessions. At follow-up, treatment satisfaction was measured by one question with a 5-point ordinal response scale ranging from “very satisfied” to “very dissatisfied.” This single item has been validated on patients with low back pain in a Norwegian clinical setting [24]. Use of other healthcare modalities was also assessed by self-report at the follow-up; frequency and type of healthcare provider were included here (none, general practitioner, specialist, chiropractor, physiotherapist, manual therapist, other providers).

### Statistical analysis

As a pilot trial, the analysis was mainly descriptive to inform the design of a full trial. A CONSORT flowchart shows the flow of participants into the pilot trial, numbers allocated to each treatment arm, numbers of follow-up responders, and number of participants included in the analysis. Feasibility in terms of selection criteria, recruitment and follow-up rates, and adherence to and experiences by the GDS treatment are presented descriptively. Descriptive statistics were used to summarize background and clinical variables at baseline, which are presented for the two treatment arms. Descriptive statistics, including normality test, were also used to summarize the four key clinical outcomes for each treatment arm: continuous variables are presented with mean and standard deviation (SD) and median and interquartile range (IQR) due to the small sample size. Categorical variables are presented as proportions and percentages. The degree of missing data to the four outcome measures is also reported. The distribution of baseline and follow-up scores of the four outcome measures was visually inspected by distribution plots. The mean change in outcome scores from baseline to 4 months was calculated for each treatment arm along with associated 95% confidence intervals (CI). When normally distributed, the mean difference between the two treatment arms for the four outcome measures was analyzed by analysis of covariance (ANCOVA), adjusting for the baseline scores in the outcome measures. The mean difference between the two treatment arms and the associated 95% CI in the four outcome measures was used to inform the optimal choice of a primary outcome for a full trial. This also included the amount of missing data at the item and scale levels, any evidence of floor or ceiling effects, the precision of the outcome measures based on the standard error of measurement, and their responsiveness to change.

In order to calculate number needed to treat (NNT) for sample size calculations for a future full-scale trial, the proportions of participants achieving a *minimal important change (MIC) in disability* were calculated for the ODI. The MIC for the ODI is estimated to be a change of 8 to 10 points on the 0–100 scale for Norwegian patients undergoing surgery due to spinal stenosis [25] or disc herniation [17]. By dichotomizing the ODI change score to 8 or more versus less than 8, we calculated the NNT by estimating the absolute risk reduction (ARR) in the intervention versus the control group and dividing 1 by this estimate (*NNT* = 1/ARR) [26, 27]. IBM SPSS version 26 (IBM Corporation, Armonk, NY, USA) was used in the statistical analyses.

## Results

### Process of recruitment, eligibility, and willingness to participate

**Fig. 1:**
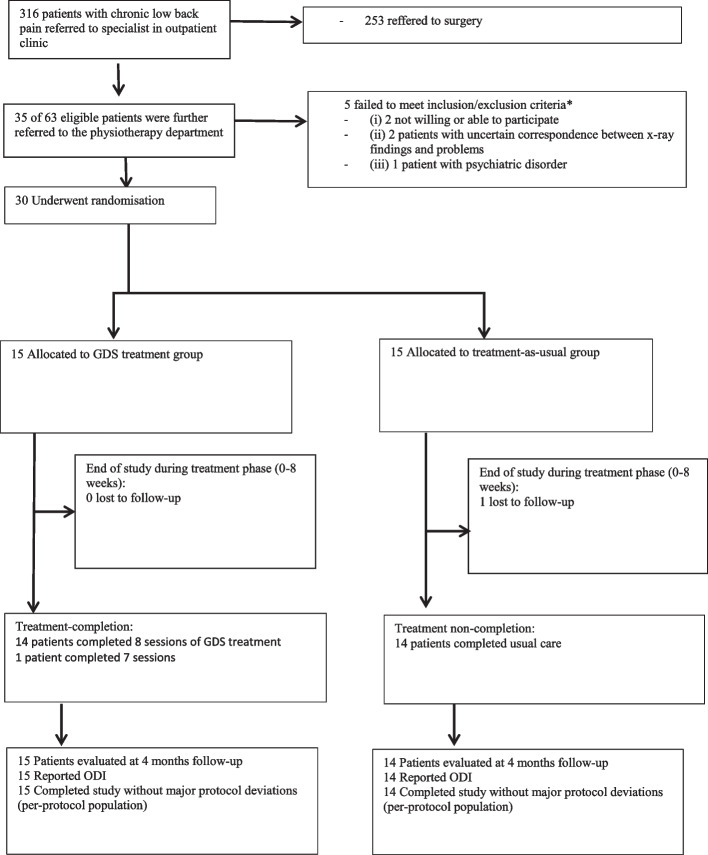
Participants flow through the pilot study

During a period of 19 months (from 15 November 2018 to 03 July 2020), a total of 316 patients with chronic LBP were referred to and consulted an orthopedic surgeon or physical medicine doctor at Vestfold Hospital (estimated numbers from the hospital administration). Most of them, 253 patients, were further referred to surgery, leaving 63 eligible participants for the current pilot project. Of the 63 eligible participants, 35 participants were referred further to the physiotherapy department for information and possible inclusion, and 30 of these were willing to participate and be randomized into one of the two treatment arms (Fig. 1). The last follow-up in this trial was performed 4 months after randomization. All patients completed the follow-up, except for one patient in the control arm (reason was not provided) (Fig. 1).

### Selection criteria for a full-scale trial

The mean age at baseline of the included patients was 58 years (*SD* 9.7), and 50% were males. Most of the participants had lower levels of education (80%), did not smoke (86%), and were married (77%), and 37% were not working. Most of the patients had a verified diagnosis of spinal stenosis without spondylolisthesis, whereas between approximately 16% had spinal stenosis with degenerative spondylolisthesis. Furthermore, 40% of the patients reported the actual pain episode enduring more than 2 years, and 54% used pain medication weekly or more frequently. Only a minor proportion reported use of sleep/relaxation medication weekly or more often (13%) and had a high risk for persistent disabling pain according to the STaRt screening tool (17%). Table 1 shows baseline characteristics of participants allocated to the intervention and control group. The randomization led to equal groups with respect to most of the baseline variables except for radiating pain to the buttocks and/or thighs and use of pain medication. A higher proportion of patients reported radiating pain to the buttocks and/or legs in the intervention group as in the control group (60% vs 27%), and more patients in the control group used pain medication. Likewise, the patients in the GDS arm reported a higher baseline score in leg pain as compared to the control arm, with a mean score of 69 versus 49, respectively (or a median score of 70 vs 20) (see Table 2). The scores in back-related disability by the ODI, in back pain, and health-related quality of life were similar across the two arms (Table 2).

**Table 1:** Baseline characteristics of participants randomized to the Godelieve Denys-Struyf (GDS) treatment arm and treatment as usual (control) arm

	GDS (***n*** = 15)	Treatment as usual (***n*** = 15)
Age, years, mean (SD)	59.0 (9.5)	57.5 (10.2)
Women, *n* (%)	7 (47)	8 (53)
Educational level, *n* (%)		
Primary or high school (12 years)	8 (53)	9 (60)
College or university (< 4 years)	4 (27)	3 (20)
University (≥ 4 years)	3(20)	3 (20)
Smoking, *n* yes (%)	2 (13)	2 (13)
Marital status, *n* (%)		
Married	13 (87)	10 (67)
Cohabitant	1 (7)	0
Single	1 (7)	5 (33)
Employment status, *n*		
Employed	6 (40)	5 (33)
Sick leave	4 (27)	4 (27)
Disability pension	4 (27)	3 (20)
Unemployed	0	2 (13)
Age pensioned	5 (33)	3 (20)
Pain localization, *n* (%)		
Back pain	5 (33)	9 (60)
Radiating pain to the buttocks and/or thighs	9 (60)	4 (27)
Sensibility changes in back/buttocks/thighs	1 (7)	2 (13)
Diagnosis, *n* (%)		
Spinal stenosis without spondylolisthesis	9 (60)	8 (54)
Spinal stenosis with spondylolisthesis	3 (20)	2 (13)
Other degenerative disc disease	3 (20)	5 (33)
Former disc surgery^a^	3 (20%)	3 (20%)
Pain duration, *n* (%)		
3–12 months	3 (20)	8 (54)
12–24 months	6 (40)	1 (7)
> 2 years	6 (40)	6 (40)
Use pain medication weekly or more, *n* (%)	7 (47)	9 (60)
Use sleep/relaxation medication weekly or more, *n* (%)	2 (13)	2 (13)
STaRt Back risk groups, *n* (%)		
Low	5 (33)	6 (40)
Moderate	8 (53)	6 (40)
High	2 (13)	3 (20)

^a^Not spinal fusion surgery (exclusion criteria)

**Table 2:** Descriptive analysis of four clinical outcomes for participants in the Godelieve Denys-Struyf (GDS) arm and treatment as usual (TAU) arm with data at baseline and 4-month follow-up

Outcomes	Baseline		4-month follow-up		Mean difference between groups, GDS vs TAU (95% *CI*)^d^
	GDS (*n* = 15)	TAU (*n* = 14)	GDS	TAU	
ODI^a^ (0–100), mean (SD)	32.9 (11.0)	35.6 (11.4)	24.3 (12.5)	38.6 (14.5)	-13.1 (-22.2 to -3.9)
Median (IQR)	30 (10)	36 (16)	24 (22)	36 (25)	
Back pain^b^ (0–100), mean (SD)	63.1 (14.2)	53.0 (17.3)	33.6 (24.5)	59.7 (23.8)	-33.9 (-50.7 to -17.0)
Median (IQR)	70 (20)	50 (30)	40 (49)	70 (35)	
Leg pain^b^ (0–100), mean (SD)	69.0 (13.8)	48.7 (22.3)	35.5 (26.7)	58.6 (24.5)	-37-0 (-56.1 to -18.0)
Median (IQR)	70 (19)	50 (40)	20 (55)	60 (33)	
EQ5D-5L^c^, mean (SD)	0.63 (0.22)	0.61 (0.25)	0.73 (0.13)	0.58 (0.29)	-0.14 (-0.31 to 0.03)
Median (IQR)	0.73 (0.40)	0.73 (0.67)	0.75 (0.22)	0.68 (0.47)	

^a^*ODI* Oswestry Disability Index (scored from 0 to 100), higher scores indicate more severe pain and disability

^b^*VAS* visual analogue scale (scored from 0 to 100), higher scores indicate more pain

^c^*EQ5D-5L* EuroQol’s health-related quality of life, 5L version (scored from −0·59 to 1), higher scores indicating better quality of life

^d^The mean difference estimates between groups are adjusted for baseline score of the outcomes

### Participant’s compliance to and experience with GDS treatment

All participants received the intended treatment based on the allocation. In the GDS arm, 14 patients received 8 treatment sessions, and one patient had 7 sessions (Fig. 1). The participants complied with the principles in the GDS treatment. Most of them, 11 (73%) reported to be very satisfied with the treatment, and 4 were slightly satisfied. No adverse events or poor experiences were reported, and no unintended consequences were revealed.

### Use of health care in the control group

In the control arm, two patients consulted a general practitioner (GP), two received physiotherapy, one chiropractic treatment, one alternative treatment (reflexology), and 9 participants reported no treatment during the follow-up.

### Clinical outcome measures and scores

There were no missing data for the outcome measures at baseline and follow-up. No floor or ceiling effects were shown in the total score of either ODI or the EQ5D-5L. There were large improvements in mean change scores in both the ODI and in pain intensity in back and leg pain (Table 2), whereas there was a deterioration in these scores in the control arm. There were minor changes in the EQ5D scores in both treatment arms (Table 2). The mean difference estimates, adjusting for the baseline scores of the outcome measure, were significantly larger (both statistically and clinically) in the GDS arm compared to the control arm with treatment as usual, suggesting the effects are of clinical interest and worthwhile to pursue in a future full-scale trial (Table 2).

### Numbers needed to treat

Achievement of a MIC, based on recommended cut-off value of 8 points in the ODI, occurred in 7 out of 15 patients (47%) in the GDS group, whereas in the control group, none of the patients achieved this amount of improvement. This gives a number needed to treat (NNN) of 3.14, meaning that we need to treat three patients with GDS treatment in order to achieve a MIC, in this case an 8-point reduction to the ODI score. Bender’s 95% confidence interval around this estimate was wide however (due to small sample size), ranging from 1.90 to 18.82.

## Discussion

This pilot trial showed that the feasibility in terms of eligibility criteria, patient information, processes for consent and randomization, follow-up rate (short term), treatment outcomes, treatment protocol, and compliance to the GDS intervention worked well. The recruitment rate was slow, however. Furthermore, there was a substantial improvement in back-related disability and pain in the GDS treatment group, whereas there was a minor deterioration in the control group. These differences are interesting and would be worthwhile testing out in a full-scale trial.

There are however minor adjustments for a full-scale trial to consider. First, the recruitment procedure took longer time than expected. The main reason was probably that the doctors often forgot to send eligible patients to the physiotherapy department and needed frequent reminders of our study. Given that the vast majority of referred eligible participants were willing to participate, and willing to be allocated either to the GDS intervention or control intervention by chance, it is possible to conduct a more rapid recruitment. This can be done by inviting all patients referred to an orthopedic examination with chronic LBP and lumbar disc degeneration to meet with a physiotherapist in the project group, regardless of planning surgery or not. Taking into consideration the costs and the risk of adverse events in surgery, these patients could be recommended to try GDS treatment before moving on with surgery. The GDS treatment is considered safe [14, 15], and no adverse events were reported in our study. In contrary, the patients reported to be highly content with this treatment and achieved a substantial improvement in three out of four outcome measures as compared to the participants receiving treatment as usual.

Another adjustment for a full-scale trial concerns the difference in leg pain between the two groups in this pilot trial. People with LBP and radicular pain or radiculopathy are often more severely affected and have poorer treatment outcomes as compared to those with back pain only [28]. Therefore, in a full-scale trial, one should consider a stratified randomization procedure, which will ensure equal distribution of patients with leg pain in the two arms.

We believe that the number of GDS treatment sessions was optimal even though some patients would have preferred even more treatments. The participants were encouraged to follow a few principles of movement and to conduct some stretching exercises at home, twice a week or when they felt that their body needed it. On the other hand, a 1-h session of individual GDS treatment is longer than most other physiotherapy sessions. An advantage by this long session is that it gave an opportunity to have a thorough dialogue with the participants, where they often opened up regarding different topics, e.g., previous treatment experiences, how they felt to be constantly searching for effective treatment, and the fear of becoming more disabled than in the current situation. A 1-h session also gave the physiotherapist the opportunity to explore which type of movements the participants tolerated and to adjust treatment and dosage according to the response from the participants.

The large difference in back-related disability and pain intensity scores after treatment in the GDS group as compared to the control group must be interpreted carefully, as most of the patients in the control group did not seek any treatment during the 4 months after randomization. Therefore, in a full-scale trial, the optimal design would be to include a placebo group in addition to a treatment as usual and GDS group.

Although it is beyond the scope of any pilot study to claim findings that are generalizable, it is interesting to compare the differences between the two groups in posttreatment scores for the ODI and pain scales from the present pilot trial to findings from other relevant full-scale trials. A mean difference of 13 ODI points (on a 0–100 scale) is a considerable larger mean difference than what was reported in the two previous trials comparing the effectiveness of routine physical therapy and GDS treatment provided for people with subacute and chronic LBP [14, 15]. A mean difference of 13 ODI points is also larger than in several other trials, which have evaluated the effect of other types of motor control exercises for chronic nonspecific LBP on disability [11–13]. In a previous Norwegian trial on patients with severe lumbar disc degeneration, in which disc replacement surgery was compared against multidisciplinary rehabilitation, they reported a mean difference of 8.9 ODI points (95% *CI* 4.8 to 13.0) at 12 months and 6.9 ODI points (2.2 to 11.6) at 24 months in favor of disc replacement [29]. It should be noted though that the 95% CI around our 13 ODI points mean difference was wider than the 95% CI in the disc replacement trial [29]. The wide confidence intervals around our NNN estimate need to be considered in a sample size calculation for a future full-scale trial.

### Considerations and limitations

The main strength of this study is that we adhered to the CONSORT 2010 extended guidelines to randomized pilot and feasibility trials [16]. The findings suggest that in a future full trial, one needs to make adjustments concerning recruitment strategy and using a stratified design in order to ensure equal groups. Also, when calculating sample size based on our pilot results, the wide confidence intervals around the NNN estimate should be acknowledged as well as taking into account a higher rate of dropout of patients followed over a longer period in a future full-scale RCT. The main limitation is the lack of insight in the process around referring potential participants after the initial clinical consultation with a specialist at the hospital and a short follow-up period (4 months after treatment allocation).

## Conclusion

This pilot trial showed that a future full-scale trial for evaluating the effectiveness of GDS treatment for patients with chronic LBP and lumbar disc degeneration is feasible. Amendments for a future trial is to get a more efficient recruitment in place ensuring access to all eligible participant and also stratify for radiating pain to buttock and/or leg ensuring comparable groups at baseline for this variable.

## Supplementary Information

**Additional file 1.**

## Data Availability

The datasets used and/or analyzed during the current study are available from the corresponding author on reasonable request.

## Abbreviations

ANCOVA: Analysis of covariance
BMI: Body mass index
CI: Confidence interval
GP: General practitioner
IQT: Interquartile range
VHT: Vestfold Hospital Trust
LBP: Low back pain
MIC: Minimal important change
NNT: Number needed to treat
ODI: Oswestry Disability Index
RCT: Randomized controlled trial
