# Supplementary material for: Effect of Godelieve Denys-Struyf (GDS) muscle and articulation chain treatment on clinical variables of patients with chronic low back pain and lumbar disc degeneration: a pilot feasibility randomized controlled trial
Source: Pilot Feasibility Stud. 2023 Mar 17;9:44. doi: 10.1186/s40814-023-01268-4 (PMC10022086; doi:10.1186/s40814-023-01268-4)
Supplement: Supplementary file 1 — Additional file 1. [file 40814_2023_1268_MOESM1_ESM.pdf]

Table 2
CONSORT checklist of information to include when reporting a pilot trial

Section/topic and item No	Standard checklist item	Extension for pilot trials	Page No where item is reported
Title and abstract			
1a	Identification as a randomised trial in the title	Identification as a pilot or feasibility randomised trial in the title	Page 1
1b	Structured summary of trial design, methods, results, and conclusions (for specific guidance see CONSORT for abstracts)	Structured summary of pilot trial design, methods, results, and conclusions (for specific guidance see CONSORT abstract extension for pilot trials)	Page 2 and 3 (1st and 2nd paragraph page)
Introduction			
Background and objectives:			
2a	Scientific background and explanation of rationale	Scientific background and explanation of rationale for future definitive trial, and reasons for randomised pilot trial	Page 4, 5, and 6
2b	Specific objectives or hypotheses	Specific objectives or research questions for pilot trial	Last paragraph page 6
Methods			
Trial design:			
3a	Description of trial design (such as parallel, factorial) including allocation ratio	Description of pilot trial design (such as parallel, factorial) including allocation ratio	Trial design: Middle paragraph page 7<br>Allocation ratio: last paragraph page 8

Section/topic and item No	Standard checklist item	Extension for pilot trials	Page No where item is reported
3b	Important changes to methods after trial commencement (such as eligibility criteria), with reasons	Important changes to methods after pilot trial commencement (such as eligibility criteria), with reasons	First paragraph page 17
Participants:			
4a	Eligibility criteria for participants		Last paragraph page 7, continues on page 8
4b	Settings and locations where the data were collected		Mid paragraph page 7
4c		How participants were identified and consented	Mid paragraph page 8
Interventions:			
5	The interventions for each group with sufficient details to allow replication, including how and when they were actually administered		First paragraph page 10
Outcomes:			
6a	Completely defined prespecified primary and secondary outcome measures, including how and when they were assessed	Completely defined prespecified assessments or measurements to address each pilot trial objective specified in 2b, including how and when they were assessed	2nd paragraph page 11

Section/topic and item No	Standard checklist item	Extension for pilot trials	Page No where item is reported
6b	Any changes to trial outcomes after the trial commenced, with reasons	Any changes to pilot trial assessments or measurements after the pilot trial commenced, with reasons	
6c		If applicable, prespecified criteria used to judge whether, or how, to proceed with future definitive trial	1st paragraph in Discussion, page 17.
Sample size:			
7a	How sample size was determined	Rationale for numbers in the pilot trial	Last paragraph page 9
7b	When applicable, explanation of any interim analyses and stopping guidelines		Not applicable
Randomisation:			
Sequence generation:			
8a	Method used to generate the random allocation sequence		Last paragraph page 8
8b	Type of randomisation; details of any restriction (such as blocking and block size)	Type of randomisation(s); details of any restriction (such as blocking and block size)	Last paragraph page 8
Allocation concealment mechanism:			
9	Mechanism used to implement the random allocation sequence (such as sequentially numbered containers), describing any steps		Last paragraph page 8

Section/topic and item No	Standard checklist item	Extension for pilot trials	Page No where item is reported
	taken to conceal the sequence until interventions were assigned		
Implementation:			
10	Who generated the random allocation sequence, enrolled participants, and assigned participants to interventions		First and 2nd paragraph page 9
Blinding:			
11a	If done, who was blinded after assignment to interventions (eg, participants, care providers, those assessing outcomes) and how		First and 2nd paragraph page 9
11b	If relevant, description of the similarity of interventions		Not relevant
Analytical methods:			
12a	Statistical methods used to compare groups for primary and secondary outcomes	Methods used to address each pilot trial objective whether qualitative or quantitative	Second paragraph page 12
12b	Methods for additional analyses, such as subgroup analyses and adjusted analyses	Not applicable	Not applicable
Results			
Participant flow (a diagram is strongly recommended):			

Section/topic and item No	Standard checklist item	Extension for pilot trials	Page No where item is reported
13a	For each group, the numbers of participants who were randomly assigned, received intended treatment, and were analysed for the primary outcome	For each group, the numbers of participants who were approached and/or assessed for eligibility, randomly assigned, received intended treatment, and were assessed for each objective	Last paragraph page 13 + Figure 1 (additional file)
13b	For each group, losses and exclusions after randomisation, together with reasons		Page 14
Recruitment:			
14a	Dates defining the periods of recruitment and follow-up		Last paragraph page 13
14b	Why the trial ended or was stopped	Why the pilot trial ended or was stopped	The trial was not stopped
Baseline data:			
15	A table showing baseline demographic and clinical characteristics for each group		Table 1
Numbers analysed:			
16	For each group, number of participants (denominator) included in each analysis and whether the analysis was by original assigned groups	For each objective, number of participants (denominator) included in each analysis. If relevant, these numbers should be by randomised group	Table 1 and 2
Outcomes and estimation:			

Section/topic and item No	Standard checklist item	Extension for pilot trials	Page No where item is reported
17a	For each primary and secondary outcome, results for each group, and the estimated effect size and its precision (such as 95% confidence interval)	For each objective, results including expressions of uncertainty (such as 95% confidence interval) for any estimates. If relevant, these results should be by randomised group	Last paragraph page 15 and Table 2
17b	For binary outcomes, presentation of both absolute and relative effect sizes is recommended	Not applicable	Not applicable
Ancillary analyses:			
18	Results of any other analyses performed, including subgroup analyses and adjusted analyses, distinguishing prespecified from exploratory	Results of any other analyses performed that could be used to inform the future definitive trial	Not applicable
Harms:			
19	All important harms or unintended effects in each group (for specific guidance see CONSORT for harms)		Second paragraph page 15
19a		If relevant, other important unintended consequences	Second paragraph page 15
Discussion			
Limitations:			

Section/topic and item No	Standard checklist item	Extension for pilot trials	Page No where item is reported
20	Trial limitations, addressing sources of potential bias, imprecision, and, if relevant, multiplicity of analyses	Pilot trial limitations, addressing sources of potential bias and remaining uncertainty about feasibility	Page 17 and 2nd paragraph page 19
Generalisability:			
21	Generalisability (external validity, applicability) of the trial findings	Generalisability (applicability) of pilot trial methods and findings to future definitive trial and other studies	Last paragraph page 18
Interpretation:			
22	Interpretation consistent with results, balancing benefits and harms, and considering other relevant evidence	Interpretation consistent with pilot trial objectives and findings, balancing potential benefits and harms, and considering other relevant evidence	2nd paragraph page 18
22a		Implications for progression from pilot to future definitive trial, including any proposed amendments	2nd paragraph page 18
Other information			
Registration:			
23	Registration number and name of trial registry	Registration number for pilot trial and name of trial registry	1st paragraph page 7
Protocol:			
24	Where the full trial protocol can be accessed, if available	Where the pilot trial protocol can be accessed, if available	Can be provided on request (the protocol was submitted to the Ethics committee)
Funding:			

Section/topic and item No	Standard checklist item	Extension for pilot trials	Page No where item is reported
25	Sources of funding and other support (such as supply of drugs), role of funders		1st paragraph page 21
26		Ethical approval or approval by research review committee, confirmed with reference number	1st paragraph page 7 + page 20
